# Eating late in the evening is associated with childhood obesity in some age groups but not in all children: the relationship between time of consumption and body weight status in U.S. children

**DOI:** 10.1186/1479-5868-6-27

**Published:** 2009-05-21

**Authors:** Stephanie Eng, David A Wagstaff, Sibylle Kranz

**Affiliations:** 1 Department of Nutritional Sciences, College of Health and Human Development, The Pennsylvania State University, 5G Henderson Building, University Park, Pennsylvania 16802, USA; 2 Human and Health Development Consulting Group, College of Health and Human Development, The Pennsylvania State University, 153 South Henderson Building, University Park, Pennsylvania 16802, USA; 3 Department of Nutrition and Dietetics, East Carolina University, Rivers Building, Greenville, North Carolina 27858, USA; 4 Department of Nutritional Sciences, The Pennsylvania State University. 331 Rivers Building, Greenville, North Carolina, USA

## Abstract

**Background:**

Some studies in adults indicate a positive correlation between eating later in the day and overall energy intake as well as body weight status. Thus, the time of food intake may be a risk factor in childhood obesity. This study was designed to describe the proportion of energy consumed in the time from 4 pm to midnight measured in two-hour increments and to determine a potential association between the time of proportion of energy consumed and body weight status.

**Methods:**

Dietary, anthropometric, and socio-demographic data of 2–18 year olds (N = 11,072) of the National Health and Nutrition Examination Survey (NHANES) 1999–2004 was examined to describe the proportion of total energy consumed within two-hour time periods between 4 pm and midnight. To examine the potential association between eating later in the day and body weight status, generalized estimating equations (GEE) models were used to quantify the effect of time trends (proportion of total energy consumed in each 2-hour time period from 4 pm to 11.59 pm) on body weight status. Analysis was conducted in the total sample and in subgroups stratified by sex, ethnic group (Non-Hispanic white, Non-Hispanic black, Mexican American, Other Hispanic, and Other Race including multi-racial) and age group (2–5, 6–11, and 12–18 year olds). Complex sample survey analysis were used to assess differences at a significance level of p-value < 0.05.

**Results:**

Proportion of energy consumed varied by sex, ethnic group, and age groups between 4 pm and 11.59 pm. Compared to healthy weight children, overweight school-age children consumed significantly higher while overweight adolescents consumed significantly lower proportions of total daily energy with each advancing two-hour time increment.

**Conclusion:**

The association between the circadian rhythm of eating and body weight status needs to be investigated further to examine the effect of time of consumption on the risk of childhood obesity. Especially longitudinal studies in diverse child populations would help elucidate the importance of time of eating on obesity.

## Background

According to most recent prevalence estimates [1], adult obesity rates increased in 37 states of the United States (U.S.) in the past year. Rates increased for a second consecutive year in 24 states and for a third consecutive year in 19 states. None of the states incurred decreasing obesity rates. To date, an estimated two-thirds of American adults and 23 million children are either overweight or obese. Thus, efforts in childhood obesity prevention need to be intensified.

Studies on childhood obesity include the investigation of the relationship between non-modifiable characteristics such as age, ethnicity, and sex as well as modifiable factors such as diet quality and nutrient composition [2]. The time of food consumption may be an additional, yet understudied, component affecting the development of childhood obesity.

As a prospective study with seven-year follow up in healthy 8–12 years old girls showed, eating later in the day (defined as intake after 5 pm and before 6 am on the following day) was associated with a small but significant positive effect on body-mass-index (BMI) z-score [3]. The findings of that study cannot be generalized to the overall pediatric population, since the sample was limited to females 8–12 years old from at least medium income, well-educated families.

To date, there is lack of data on a potential association between children's body weight status and the time of the day when energy-contributing foods are consumed. The examination of this relationship is complicated by known variations of energy intake, such as the fact that males consume larger total amounts of energy compared to females or that total daily energy intakes increase, as children get older. To accommodate these differences, the exploration of the association between the time of energy consumption and body weight status must be examined by sex and age groups.

In an effort to contribute to childhood obesity prevention efforts, the present study focused on a nationally representative sample of the U.S. pediatric population to examine the association between the proportion of total energy consumed during two-hour time periods in the afternoon and evening hours and childrens' body weight status. Regression models were fitted to the data stratified by sex and age to explore whether consuming a greater proportion of total energy intake later in the day was associated with an increased likelihood of being classified as overweight or obese. The hypothesis tested was that eating a higher proportion of total energy later in the day was associated with being classified as overweight or obese. Due to the ethnic diversity of the sample, we expected to find some energy intake reports for times not traditionally considered meal or snack times for children, such as late-night dinners in the Hispanic subpopulation.

## Methods

### Sample

The Centers for Disease Control and Prevention (CDC) conducts the National Health and Nutrition Examination Survey (NHANES, ), an ongoing survey using a multistage, stratified area design to obtain a sample of respondents that is representative of the civilian non-institutionalized U.S. population. Certain groups are over-sampled (e.g. young children, adolescents 12–19 years, African Americans, Mexican Americans, and low-income persons). Although the data are released in 2-year increments, they were designed to be merged to multi-year data sets [4]. This study uses the sociodemographic, dietary, and anthropometric data of the NHANES 1999–2004 in children ages 2–18 years old who were not breastfed and who were classified as having "reliable" intake reports (N = 11,072).

### Socio-demographic data

The adult completing the household interview during the NHANES survey reported socio-demographic information, such as age, sex, race, and ethnicity. For this study, the age variable was used to create three mutual exclusive age groups: 2–5 year old children, 6–11 year olds, and 12–18 year olds to accommodate changes in eating behavior subsequent to gaining greater independence from the care taker and enrollment in school.

Children's race was determined based on the interview responder's categorization of the child as American Indian or Alaskan Native, Asian, black or African American, Native Hawaiian or Pacific Islander, White, or other. In addition, self-reported ethnic background was determined by whether the child was Mexican American, other Hispanic or Latino, both (Mexican and other Hispanic), or not Hispanic. In an effort to capture the cultural differences in food intake behaviors of children living in the U.S., the race and ethnic background variables were used to define five mutual exclusive ethnic groups: Non-Hispanic white, Non-Hispanic black, Mexican American, Other Hispanic, and Other Race Including Multi-Racial.

### Dietary intake

NHANES dietary data were collected with interviewer administered 24-hour recalls. In the survey waves of NHANES 1999–2000 and 2001–2002, dietary intake data was collected for one single day. In the NHANES 2003–2004, two days of 24-hour recalls were collected but only the first day was considered for this study. Data from all three NHANES waves were combined and used in the analysis.

To collect 24-hour dietary recall data, respondents were asked to report all consumption of foods and beverages during the past 24 hours using a multiple-pass approach methodology [5]. Intake information included the amount and type of food consumed as well as the time of consumption. Caretakers reported the diets for children less than six years old; 6–12 year olds reported their own diet with the help of an adult if they chose. Children ages 12–18 year olds reported their diets.

A standardized protocol of dietary intake data collection ensures the high quality and consistency across dietary interviewers in the NHANES data [6]. For each food reported, the time and occasion of consumption was ascertained during the dietary intake interview. Survey respondents were asked to recall the time when food consumption began and this time was entered into the data recall software. During a second pass, interviewers repeated all foods on record and respondents were asked to either confirm or recall the time when each food was consumed. Responses were verified with probing questions, such as "was that am or pm?" or "in the morning" vs. "in the afternoon". If the respondent did not recall the exact time the food was consumed, interviewers probed for an approximate consumption time. When it was not possible to obtain an approximation of the eating time from the subject, the interviewer entered a default time according to the following guidelines: 8:00 am for breakfast, 10:00 am for morning snack, 12:00 pm (noon) for lunch, 3:00 pm for afternoon snack, 6:00 pm for dinner, and 9:00 pm for evening snack. For example, if the subject did not recall the exact time a food was consumed but remembered that he/she ate at home after returning from school, the interviewer would probe for a time by asking for an approximate time when the subject got home from school, if the food was consumed immediately upon arrival at home or somewhat later, and similar questions. If the probing did not result in a time approximation, the interviewer ascertained whether the subject would call the eating occasion an afternoon snack or rather a dinner and then entered the corresponding default time to the record.

For this study, dietary intake records were used to calculate childrens' total energy intake (kcal per day) as well as the proportion of total energy consumed within two-hour time increments (proportion of total energy consumed per two-hour period). To focus on the hypothesis of this study that eating higher proportions of total energy later in the day is associated with being classified as overweight or obese, the analysis was limited to the latter hours of the day (between 4 pm and 11.59 pm).

### Anthropometric data

Measured height and weight as well as calculated body mass index (BMI, weight in kilograms/height in meters squared) are available in the NHANES data set. Weight (kilograms) was obtained using a digital scale; standing height was measured with an electronic stadiometer and recorded in meters. CDC's BMI-for-age and sex-specific growth charts were used to create four distinct groups: underweight (less than 5^th ^percentile), healthy weight (5^th ^to 84^th ^percentile, overweight (between the 85^th ^and 94^th ^percentile), and obese (≥ 95^th ^percentile) [7].

As expected, the anthropometric data was not normally distributed, thus, the method by Cole et al. to construct a smoothed curve using the calculated power (L), mean (M), and coefficient of variation (S) to provided standards in terms of centiles was employed to determine children's normalized growth centile standards [8]. Underweight and healthy weight children were combined to one group ("healthy weight") and two binary dummy variables were created that equaled "=1" when children were classified as overweight or obese and "=0' otherwise.

### Statistical analysis

All analysis was conducted using complex sample survey routines (version 9.2; StataCorp LP, College Station, Texas, USA [9]). Descriptive statistics (means or proportions and standards errors (SE)) and Student t-test) were calculated. For visual presentation of the relationship between body weight status and proportion of energy consumed in each two-hour time increment, connected line plots were generated.

The association between body weight status and the proportion of energy consumed in advancing time periods was examined using generalized estimation equations (GEE). A four-level discreet time trend variable was created to indicate dietary energy intakes at time point zero (4 pm to 5.59 pm), one (6 pm to 7.59 pm), two (8 pm to 9.59 pm), and three (10 pm to 11.59 pm). GEE models included covariates for sex, body weight categories, ethnic group category, and age group. The Institutional Review Board (IRB) in the Office of Research Protection (ORP) at Pennsylvania State University granted approval for this study based on the use of secondary data with no person identifiers.

## Results

The study included 11,072 children two through 18 years old who provided a total of 52,506 food records. Approximately 50% of the sample was boys and 60% were non-Hispanic white (Table 1). The proportion of healthy-weight children decreased with increasing age while the proportion of overweight or obese children increased with increasing age. Basic descriptive statistics indicated that average daily total energy consumption was significantly higher in males than in females and significantly increased as children were older.

**Table 1:** Sociodemographic and body weight characteristics of the sample (in percent)

	**Age groups (years old)**			**Total**
	2–5	6–11	12–18	Sample

Sample Size (N)	2,284	2,998	5,797	11,079

Boys	50	51	51	51

Ethnic Group				

Non-Hispanic White	60	60	62	61

Non-Hispanic Black	13	15	14	14

Mexican American	14	13	11	12

Other Hispanic	6	7	7	7

Other race and multicultural	7	6	7	6

Body Weight				

Healthy weight	86	66	64	69

Overweight	11	21	21	19

Obese	6	13	15	13

On average, children classified as being healthy weight consumed 45% of their total daily energy between 4 pm and midnight. Children classified as overweight and obese consumed a significantly higher proportion of energy in the same time period (47% and 49%). Student t-test indicated that compared to their healthy weight peers, average energy consumption in overweight children was 23.0 kcal and in obese children 27.4 kcal higher between 4 pm and 8 pm (p-value < 0.001 and p-value < 0.03, respectively). No statistical difference was found in the amount of energy consumed between 8 pm and midnight among the three body weight groups.

Visual inspection of the consumption patterns does not indicate statistical significance between the patterns of energy intake in the three age groups but serves purely as illustration of the differences in energy intake habits in the latter part of the day.

**Figure 1:**
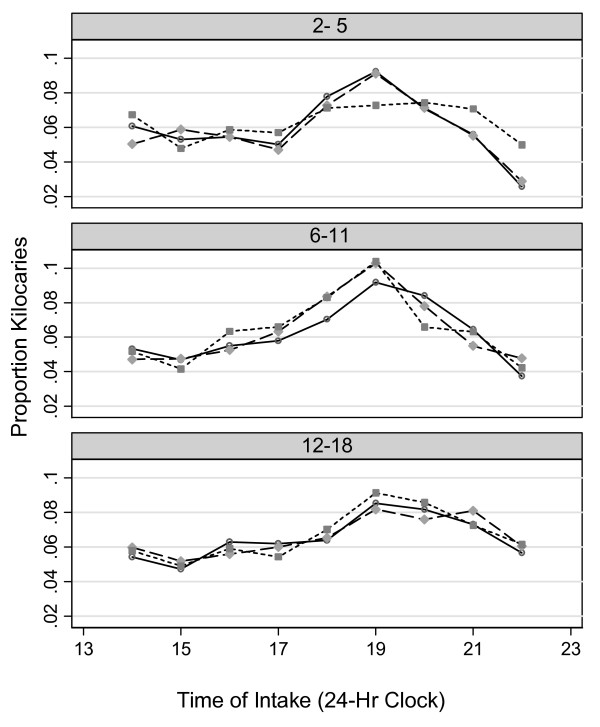
**Proportion of total energy consumed by U.S. children in the afternoon and evening hours 4 pm to midnight by age group and body weight status**. The circles represent children with healthy body weight, diamonds represent children who are classified as overweight and solid squares represent obese children.

As the line plots of Figure 1 indicate, in 2–5 year olds the proportions of total energy consumed were similar between the body weight groups until 6 pm. Intakes appear to peak for the healthy weight and overweight children at 7 pm followed by a steady decease. Although children classified as obese seemed to have lower proportions of energy at 7 pm, they maintained a higher levels of energy consumed between 8 pm and 10 pm. In 6–11 year old youths, children classified as obese and overweight seemed to have larger proportions of energy 4 pm and 7 pm than the healthy weight children. Among the 12–18 year old youths, the children in the three body weight groups seemed to have similar intake patterns. However, the line representing the proportion of energy consumed by the obese children consumed a larger proportion of energy between 6 pm and 9 pm.

To determine statistical significance of the association between body weight status and the time of energy consumption GEE fixed effects models were fitted to the data in the total sample as well as grouped by sex, age, and ethnic group. The model fitted for the total sample included the sex (dichotomous) and age (continuous) variables as well as the 4-level dummy variables for linear time trend and the variable for children's body weight status (children in the healthy weight group served as referent). Ethnic group was not a significant contributor to the model in the full sample. The stratified models for the sex, age, and ethnic groups only included the time trend and body weight status variables. Table 2 presents the beta coefficient, standard error, and p-value of proportion of energy consumed within each two-hour time period from 4 pm to 11.59 pm among children in the total combined sample as well as grouped by sex, age group, and ethnic group.

**Table 2:** Results of Generalized Equation Models (GEM) of total energy intake (kilocalories per two-hour increment) in the time 4 pm to midnight in the total sample of 2–18 year olds and by sex, ethnic groups, and age groups

	Beta Coefficient	Standard Error	P-value
Total sample population			

Sex (referent: male)	-36.3	2.79	<0.001

Year (in age)	7.8	0.31	<0.001

Overweight*	0.2	3.86	0.967

Obese*	3.0	5.08	0.552

Linear Time Trend	-2.5	0.78	<0.001

Males only			

Year (in age)	10.7	0.49	<0.001

Overweight	-5.2	5.88	0.380

Obese	0.3	7.85	0.966

Linear Time Trend	-4.4	1.17	<0.001

Females only			

Year (in age)	4.8	0.35	<0.001

Overweight	4.1	4.92	0.407

Obese	4.3	6.41	0.505

Linear Time Trend	-0.4	1.02	0.698

Non-Hispanic White			

Sex	-41.2	4.08	<0.001

Year (in age)	8.4	0.45	<0.001

Overweight	0.1	5.74	0.987

Obese	5.2	8.22	0.527

Linear Time Trend	-2.7	1.11	0.016

Non-Hispanic Black			

Sex	-33.4	3.56	<0.001

Year (in age)	7.4	0.39	<0.001

Overweight	-0.4	5.04	0.931

Obese	-0.8	5.14	0.869

Linear Time Trend	-4.0	1.03	<0.001

Mexican-Americans			

Sex	-26.5	3.36	<0.001

Year (in age)	6.6	0.37	<0.001

Overweight	1.1	4.42	0.802

Obese	-5.2	4.71	0.268

Linear Time Trend	-0.5	1.09	0.639

Other and multicultural			

Sex	-35.0	12.32	0.005

Year (in age)	6.9	1.19	<0.001

Overweight	12.3	18.62	0.507

Obese	0.7	20.28	0.973

Linear Time Trend	5.0	4.20	0.237

Other Hispanic			

Sex	-15.6	11.74	0.183

Year (in age)	5.7	1.16	<0.001

Overweight	-9.0	14.15	0.524

Obese	14.1	24.27	0.561

Linear Time Trend	-4.5	3.38	0.182

2–5 year olds			

Sex	-3.1	3.39	0.354

Year (in age)	7.2	1.56	<0.001

Overweight	-1.2	5.34	0.826

Obese	5.8	6.78	0.389

Linear Time Trend	1.4	0.98	0.141

6–11 Year olds			

Sex	-22.2	4.47	<0.001

Year (in age)	4.9	1.33	<0.001

Overweight	16.7	6.20	0.007

Obese	10.3	7.74	0.184

Linear Time Trend	-4.6	1.23	<0.001

12–18 year olds			

Sex	-66.2	5.08	<0.001

Year (in age)	7.9	1.28	<0.001

Overweight	-15.9	6.07	0.009

Obese	-5.2	8.12	0.518

Linear Time Trend	-2.9	1.40	0.039

* referent: Healthy body weight group

The tests of the regression coefficients from the GEE model in the total population indicated that there was a significant negative linear trend in energy consumption in the afternoon and evening hours (p-value < 0.001) but this decline of proportion of energy consumed with advancing time was not significantly associated with body weight status. As expected, increasing age was associated with higher energy intake (additional 8 kilocalories per year of age) and females consumed significantly less energy (42 kilocalories) than their male counterparts (p-values < 0.001).

When examining the GEE models grouped by sex, increasing age was significantly associated with higher energy intakes (11 kcal in boys and 5 kcal in girls per increase year of age, p-value < 0.001). A statistically significant negative time trend effect on energy intake was observed in boys (p-value < 0.001) but there was no association with body weight status.

The GEE model fitting the data of each of the five ethnic subsamples indicated significant lower energy intakes in girls than in boys (p-value < 0.001) in all ethnic groups but the "other Hispanic" but higher energy intakes with increasing age (p-value < 0.001). In the models of the Non-Hispanic white and Non-Hispanic black children, a significant negative time trend of energy intake was observed (p-value < 0.001).

The GEE model fitted in each one of the three age groups showed that 6–11 year old and 12–18 year old girls consumed significantly less energy than boys but increasing age was associated with higher energy intakes in all three age groups (p-value < 0.001). In children ages 6–11 and 12–18 years old statistically negative associations between energy intake and time trends were found (p-value < 0.001 and p-value < 0.039). The association between body weight status and energy intake was positive in the 6–11 year old overweight children, who consumed 17 kilocalories more than their healthy weight counterparts (p-value < 0.007) per two-hour time period while 12–18 year old overweight children consumed 16 kilocalories less per two hour time period than their healthy weight peers (p-value < 0.009). Examination of the beta-coefficient and p-values of each category in the time trend variable indicated differences in the proportion of total energy intake consumed between time period zero and one, (4 pm – 5.59 pm compared to 6 pm – 7.59 pm). There were no significant changes in the proportion of energy consumed after 8 pm in either age group.

## Discussion

Some studies conducted in adults indicated that consuming larger amounts of food in the evening hours was associated with higher body weight. In particular, researchers found that consumption of a late dinner was associated with larger overall energy intake throughout the day; conversely, individuals with larger energy intake in the morning hours tended to have smaller total energy intake [10]. Therefore, circadian variations in energy intake can be assumed to be an important modifiable dietary intake factor in obesity prevention.

In an effort to improve the understanding of a potential relationship between the time of energy consumption and the current childhood obesity epidemic in the U.S., we examined the association of the relative amount of childrens' total daily energy intake consumed in the latter part of the day and their body weight status in a nationally representative sample of 2–18 year old children. As the results of this study indicated, total energy intake varied by age and sex of the children. The proportion of total daily energy consumed in the latter part of the day was associated positively with being classified as overweight in the 6–11 year olds but negatively in the overweight 12–18 year olds. No significant differences were found between the proportion of energy consumed between 8 pm and midnight and body weight status. This lack of association however, may have been due to the limited number of individuals consuming food so late in the day (n = 3,802), which may have affected the statistical power to detect significant differences in the proportion of energy consumed between the time periods.

As reported previously, healthy weight children consume larger amounts of energy during breakfast whereas overweight and especially obese children eat less during that meal [11,12]. Our findings add to these results in that overweight school-age children also consume higher levels of energy later in the day compared to the healthy weight children. As children get older, they become more independent and not only consume larger amounts of energy but may also change the frequency of eating throughout the day. This phenomenon reflects the structure of children's life. Young children spend a considerable amount of their day in school with structured meal and snack times. It is only when children are in junior high school and high school that they can purchase food more freely at will and may opt not to eat at all during the morning and early afternoon but focus their consumption patterns to the latter part of the day.

Our study indicates a significant association between body weight status and meal or snack consumption in the latter part of the day only in school-age children and adolescents. Surprisingly, children ages 6–11 years old who were classified as overweight consumed larger proportions of total energy in the later in the day whereas overweight adolescents consumed less energy in the same time period. This apparent paradox is not easily explained. The positive association between food consumption later in the day and higher body weight status in the 6–11 year olds is more intuitive and confirm results from studies on the Night Eating Syndrome (NES), which indicate that adults who consume food during the night have higher body weights than individuals who do not eat during night time [13]. One of the underlying mechanisms for this observation could be the lack of physical activity in the evening hours or overeating due to distractions such as watching TV. The energy in meals or snacks consumed later in the day might be stored in the body fat stores, rather than being oxidated to energy for physical movement during the more active hours of the day.

A recent study investigating the characteristics of individuals with NES elucidated that the increased risk for higher body weight status may not be attributable to the NES but to the underlying psychological factors leading to NES [14]. For instance, in a study using body weight matched samples, individuals with NES were found to have altered distribution of food intake throughout the day but also reported higher prevalence of depressed mood, sleep disturbance, disordered eating, perceived stress, decreased quality of life and increased anxiety.

Results of this study are limited by the use of only one-day dietary intake estimates rather than multiple-day food records to estimate average energy intake. However, since the focus of this paper was to examine dietary intake patterns and report on the association between the time of energy consumption and body weight status in the U.S. pediatric population, the use of a single 24-hour recall was appropriate. In addition, the dietary intake data of the sample is limited by the use of a proxy for the diets of young children, which were reported by their caretakers due to the cognitive limitations in individuals less than six years old. Although this procedure to obtain dietary intake data on young children is widely accepted as the most appropriate method, it is limited by the potential for under- or over-reporting of intakes based on the caretakers' characteristics and the likelihood that the caretaker did not personally observe all food intake occasions of the young children.

This study was designed to increase the understanding of the potential importance of the circadian rhythm of food intake on childhood obesity. Heretofore, no data was available on the effect of the time of energy consumption on childhood obesity in sex, age, and ethnic subgroups of the U.S. pediatric population. Although this study was of purely observational nature, results aid in the generation of hypothesis for future studies on childhood obesity treatment and prevention efforts.

## Conclusion

Increasing age, sex, and belonging to certain ethnic groups affects food consumption patterns in children 2–18 years old. Results of this study indicate a need for further in-depth exploration of the effect of the proportion of total energy consumed during the day and especially in the afternoon and evening hours on childhood obesity development. Longitudinal studies using large samples of children from various ethnic backgrounds and age groups would contribute substantially to the body of knowledge on the potentially important effects of the time of food consumption on chronic disease development.

## Competing interests

The authors declare that they have no competing interests.

## Authors' contributions

SK conceived of this study and led the analysis and manuscript preparation. DAW contributed to the manuscript by programming the statistical analyses and provided data analytic advice to SK. SE, an undergraduate student of the Schreyer's Honors College participated in the interpretation of results and preparation of the manuscript in fulfillment of the requirement for her honor's thesis.
